# Hsa-miR-31-3p targets CLDN8 to compromise skin barrier integrity in psoriasis

**DOI:** 10.1016/j.bbrep.2025.101976

**Published:** 2025-03-13

**Authors:** Yunhua Tu, Li Wang, Lijun An, Li He

**Affiliations:** a Department of Dermatology, First Affiliated Hospital of Kunming Medical University, Kunming, 650032, China; b Department of Dermatology, The Second People's Hospital of Guiyang, Guizhou, 550081, China

**Keywords:** Psoriasis, Skin barrier, Hsa-miR-31-3p, CLDN8, Tight junction

## Abstract

Skin barrier dysfunction in psoriasis has emerged as a significant concern, yet the underlying molecular mechanisms remain incompletely understood. This study investigates the role of hsa-miR-31-3p in regulating skin barrier function through its interaction with claudin-8 (CLDN8) in psoriasis. Through analysis of clinical samples and public datasets, we observed significantly impaired skin barrier function in psoriasis patients, characterized by increased transepidermal water loss and decreased stratum corneum hydration. Notably, CLDN8 expression was markedly downregulated in psoriatic lesions, while hsa-miR-31-3p levels were elevated. Bioinformatics analysis and molecular studies revealed that hsa-miR-31-3p directly targets the 3′UTR of CLDN8, leading to its downregulation. In vitro experiments demonstrated that both CLDN8 knockdown and hsa-miR-31-3p overexpression compromised the permeability barrier in keratinocytes. Furthermore, in an imiquimod-induced psoriasis mouse model, administration of mmu-miR-31-3p antagomir effectively ameliorated skin barrier damage, reduced inflammatory manifestations, and restored CLDN8 expression. These findings unveil a novel mechanism whereby hsa-miR-31-3p regulates skin barrier function through CLDN8 in psoriasis, suggesting potential therapeutic strategies targeting this pathway for psoriasis treatment.

## Highlights

• Study reveals new psoriasis mechanism: hsa-miR-31-3p/CLDN8 axis linked to skin barrier dysfunction.
• Psoriasis lesions show high hsa-miR-31-3p, causing CLDN8 downregulation, a key tight junction protein.
• CLDN8 downregulation damages skin barrier, driving psoriasis development and progression.
• Targeting hsa-miR-31-3p/CLDN8 pathway could lead to new treatments for psoriasis patients.
• Research provides in vitro and in vivo evidence, supporting its potential for clinical translation.

## 1 Introduction

Psoriasis, a persistent autoimmune skin condition, manifests as distinct red patches covered with silvery scales. This global affliction shows no gender bias and currently affects approximately 60 million individuals worldwide [1,2], Its prevalence varies significantly across regions, with East Asia reporting 0.1 % and Western Europe 1.5 %, peaking in high-income nations [3]. The rising incidence of psoriasis considerably impacts patients' well-being and quality of life [4].

Despite extensive research, the exact etiology of psoriasis remains elusive. Current understanding points to a complex interplay of genetic predisposition, innate and adaptive immune dysregulation, and environmental factors [5]. Recent investigations have highlighted the significance of epidermal barrier dysfunction, microbial infections, and microbiome imbalances in psoriasis pathogenesis [6,7].

The skin's barrier function, crucial for defense against external threats, plays a pivotal role in various dermatological conditions and immunological imbalances [8]. Tight junctions, key structural components of this barrier, regulate epidermal permeability through the anchoring of membrane proteins and lipids [9]. The claudin protein family, integral to tight junctions, significantly influences epidermal permeability and is implicated in numerous skin disorders [10]. It is well-known that psoriasis is associated with significant skin barrier dysfunction, but the underlying mechanisms remain incompletely understood. Recent studies have reported that tight junction (CLDN8) and lipid biosynthesis and metabolism (FA2H and ALOXE3) products are significantly downregulated in both atopic dermatitis and psoriasis [11]. RNA sequencing has demonstrated the downregulation of tight junction and stratum corneum proteins in the skin of patients with severe allergic contact dermatitis after exposure to *p*-Phenylenediamine. Specifically, the mRNA expression of Claudin-1, CLDN8, CLDN11, CXADR-like membrane protein, occludin, membrane-associated guanylate kinase inverted 1, and MAGI2 was downregulated in patients with severe allergic contact dermatitis [12]. Consistent with these findings, our analysis of psoriasis transcriptome sequencing datasets revealed that CLDN8 is significantly downregulated in psoriatic lesions, ranking 17th among differentially expressed genes with low expression (LogFC = −2.36, *P<0.05*). This suggests that CLDN8 may play an important role in skin barrier dysfunction in psoriasis. However, how CLDN8 is regulated and its specific impact on the skin barrier in psoriasis remains unclear.

MicroRNAs (miRNAs) play a critical role in the post-transcriptional regulation of gene expression [13,14]. They function by binding to complementary sequences in the 3′ untranslated regions (3′UTRs) of target mRNAs, leading to mRNA degradation or translational repression [15]. This regulatory mechanism allows miRNAs to fine-tune the expression of thousands of genes, influencing a wide range of biological processes, including cell proliferation, differentiation, apoptosis, and immune responses [16,17]. Recent study showed that miR-155 and miR-146a exhibit abnormal expression in inflammatory skin diseases such as atopic dermatitis and psoriasis. By modulating the expression of inflammation-related genes, they influence the intensity and duration of inflammatory responses [18,19]. Further, Our analysis of public datasets revealed a significant upregulation of hsa-miR-31-3p in psoriatic tissues (Ranked first in high differential expression, LogFC = 5.78, *P<0.05*). This miRNA has been associated with diverse biological processes, including inflammation and immune regulation [20]. For instance, hsa-miR-31-3p is up-regulated during the skin wound healing process and promotes wound repair by regulating genes related to cell migration, proliferation, and matrix remodeling [21]. NF-κB activation directly inhibits PPP6C expression through the induction of miR-31, thereby enhancing keratinocyte proliferation [22]. In this study, Bioinformatics predictions suggest that hsa-miR-31-3p may target the 3′UTR of CLDN8. As mentioned earlier, CLDN8 expression is significantly reduced in psoriatic lesions. Based on these findings, we speculate that hsa-miR-31-3p may likely influence the skin barrier in psoriasis by regulating CLDN8.

This study investigates the potential mechanism by which hsa-miR-31-3p may impair skin barrier function in psoriasis through its interaction with CLDN8. Our findings suggest that hsa-miR-31-3p downregulates CLDN8 expression by binding to its 3′UTR, potentially contributing to skin barrier dysfunction in psoriasis patients. These insights offer new perspectives on the pathogenesis of psoriasis and may inform novel therapeutic strategies targeting the miRNA-mediated regulation of skin barrier function.

## 2 Materials and methods

### 2.1 Tissue specimens

The study utilized tissue samples from 29 psoriasis patients, obtained from paraffin-embedded specimens, and 31 normal tissue samples collected during orthopedic or ophthalmic surgeries [Psoriasis patients and control samples was conducted over 12 months (January 2023–December 2023)]. All surgical procedures were conducted at the First Affiliated Hospital of Kunming Medical University (Yunnan, China), with approval from the institutional ethics committee. Patient selection adhered to established psoriasis diagnostic criteria. Exclusion criteria (For patients and control samples) encompassed individuals with chronic diseases or immune system disorders (e.g., systemic lupus erythematosus, diabetes, rheumatoid diseases), as well as those who had received immunosuppressants or corticosteroids within six weeks prior to sample collection. Ethical approval and informed consent were obtained prior to sample collection.

### 2.2 HaCaT cell culture

Human immortalized keratinocyte cell lines (HaCaT cells, maintained in our laboratory) were cultured in Dulbecco's Modified Eagle's Medium (DMEM, Gibco, USA) supplemented with 10 % fetal bovine serum (Gibco) and 1 % penicillin-streptomycin (Gibco). Cells were incubated in a humidified atmosphere containing 5 % CO_2_ at 37 °C.

### 2.3 Non-invasive detection of skin barrier function

Skin barrier function was assessed non-invasively in 29 psoriasis patients and 29 healthy controls using a Cutometer dual MPA 580 (Germany). Transepidermal water loss (TEWL) and stratum corneum hydration (SCH) were measured on psoriatic lesions and corresponding sites in healthy individuals. For murine studies, TEWL and SCH were measured on the dorsal skin of experimental mice, with normal mice serving as controls. Each mouse was examined at three distinct sites on the back.

### 2.4 Hematoxylin and eosin (H&E) staining

Tissues were fixed, paraffin-embedded, and sectioned into 4 μm slices. Sections were deparaffinized with xylene and rehydrated through an ethanol gradient as follows: xylene I (5min) → xylene II (5min) → 100 % ethanol (2min) → 95 % ethanol (1min) → 80 % ethanol (1min) → 75 % ethanol (1min) → distilled water (2min). Sections were stained with hematoxylin for 5 min, washed with running water, and differentiated in acid alcohol for 30s. After rinsing in tap water for 15min, sections were counterstained with eosin for 2min. Dehydration, clearing, and mounting were performed using the following sequence: 95 % ethanol I (1min) → 95 % ethanol II (1min) → 100 % ethanol I (1min) → 100 % ethanol II (1min) → xylene carbonate (3:1, 1min) → xylene I (1min) → xylene II (1min) → mounting with neutral resin.

### 2.5 Bioinformatics analysis

Psoriasis transcriptome sequencing datasets (GSE78097, GSE14905, and GSE13355) were obtained from the NCBI database. Principal Component Analysis (PCA) clustering, differential gene expression analysis, and KEGG pathway enrichment analysis were performed. Protein interaction networks were constructed using the STRING database. Differential miRNA expression was analyzed using the psoriasis small RNA sequencing dataset GSE145305. FunRich 3.1 software was employed to examine correlations between differentially expressed miRNAs and clinical phenotypes. The TargetScan 7.2 database was used to predict miRNA-target gene interactions [Network Construction: The interaction network was constructed using Cytoscape software (version 3.9.1). Nodes represent miRNAs or target genes, and edges represent predicted interactions based on miRNA binding sites. Analysis: Key nodes were identified based on their centrality and biological relevance]. All bioinformatics analyses were conducted using R version 4.2.0.

### 2.6 Total RNA extraction from paraffin-embedded tissue

The outer paraffin layer was trimmed from the sample, and 10 μm thick tissue sections were prepared. Four sections were transferred to a 1.5 mL centrifuge tube, and 250 μL Buffer FTL was added, followed by vortex mixing. RNA extraction was performed according to the FFPE RNA Kit (Omega, USA) instructions. Finally, RNA was eluted with 50 μL DEPC-treated water, incubated at room temperature for 3min, and centrifuged at 10,000 g for 1min.

### 2.7 Real-time quantitative PCR (RT-qPCR)

Total RNA was extracted using TRIzol reagent or from paraffin-embedded tissue as described above. cDNA synthesis was performed using an mRNA Reverse Transcription Kit (Invitrogen, Thermo Fisher Scientific, USA) for mRNA and a miRcute Plus miRNA First-Strand cDNA Kit (TIANGEN, Beijing, China) for miRNA. RT-qPCR for both miRNA and mRNA was conducted using SYBR Green (TIANGEN, Beijing, China). Specific primers are listed in the supplementary material. RT-qPCR was performed on a Rotor-Gene Q instrument (QIAGEN, Germany). Cycling conditions were as follows: initial denaturation at 95 °C for 15 min, followed by 40 cycles of 95 °C for 10 s, 56 °C for 20 s, and 72 °C for 30 s. Melting curves were analyzed for all amplification products. Relative gene expression was calculated using the 2^−ΔΔCt^ method, β-actin as the reference gene for normalization. Each experiment was independently repeated three times.

### 2.8 Oligonucleotide transfection

si-CLDN8-NC si-CLDN8-1, si-CLDN8-2, hsa-miR-31-3p mimics-NC, hsa-miR-31-3p mimics (The final concentration of the aforementioned oligonucleotides is 50 nmol), miR-31-3p inhibitor (The final concentration of the oligonucleotides is 100 nmol), mmu-miR-31-3p antagomir-NC and mmu-miR-31-3p antagomir (The dosages of the latter two murine oligonucleotides can be found in Section 2.13) were purchased from GenePharma (Shanghai, China). Keratinocytes were transfected at 70–80 % confluence using Lipofectamine 3000. Transfection efficiency was confirmed by RT-qPCR. Interference sequences are provided in the supplementary material.

### 2.9 FITC-Dextran transwell permeability assay

Keratinocytes were seeded in the upper chamber of Transwell inserts (0.40 μm pore size, USA) and cultured for 24 h. Following oligonucleotide transfection for 48 h, the medium was replaced with fresh complete medium, and cells were cultured for an additional 24 h, 48 h, or 72 h. To assess FITC-Dextran flux, 1 mL of complete medium was added to the lower chamber, and 500 μL of complete medium containing 50 μg/mL FITC-Dextran was added to the upper chamber. After 60min incubation, 100 μL of medium from the lower chamber was transferred to a 96-well plate (Corning Incorporated Costar, USA). FITC-Dextran flux was measured using a microplate reader (BioTek, USA) with excitation at 485 nm and emission at 535 nm. FITC-Dextran flux was calculated as the ratio of treated group to blank control. Each experiment was repeated three times.

### 2.10 Immunofluorescence detection

Skin tissues were fixed in 4 % paraformaldehyde and processed through washing, dehydration, clearing, waxing, embedding, sectioning, baking, deparaffinization, and rehydration. Primary antibody (anti-CLDN8, 1:50 dilution, GeneTex) was applied to the tissue and incubated overnight at 4 °C. For secondary antibody incubation, slides were washed three times in PBS (5 min each) on a decolorizing shaker. The corresponding fluorescent secondary antibody was applied and incubated for 50 min in the dark [Anti-rabbit IgG (H + L) (DyLight 680 Conjugate), diluted at 1:15000, incubated at room temperature]. For DAPI staining, slides were washed three times in PBS, and DAPI dye was applied for 3–5 min in the dark. Finally, sections were observed and imaged using a fluorescence microscope.

### 2.11 Western blot analysis

Total protein from keratinocytes or tissues was extracted using RIPA. Proteins were separated by 12 % SDS-PAGE (60 V for 30min, then 95 V for 90min) and transferred to PVDF membranes (Bio-Rad) using a semi-dry transfer method. Membranes were incubated overnight with primary antibodies (anti-CLDN8, Abcam, UK; Used at concentration of 2 μg/ml), The corresponding secondary antibody was applied and incubated for 60 min (anti-GAPDH, Abcam, UK; Used at concentration of 1 μg/ml). Following ECL kit instructions, chemiluminescence solution was applied, and target bands were visualized under UV light (General Electric Company, USA). Quantification Method: protein bands were quantified using ImageJ software. The intensity of each target band was normalized to the corresponding GAPDH band as an internal control. Analysis: relative protein expression levels were calculated as the ratio of the target band intensity to the GAPDH band intensity. Data are presented as mean ± standard deviation from at least three independent experiments.

### 2.12 Luciferase reporter gene assay

Wild-type (pmirGLO-CLDN8-WT) and mutant (pmirGLO-CLDN8-MT) CLDN8 plasmids were obtained from Promega, USA. HaCaT cells were co-transfected with hsa-miR-31-3p mimics, inhibitor, and the above plasmids for 48h using Lipofectamine 3000 (Invitrogen, USA). Luciferase activity was measured using a Dual-Luciferase Reporter Assay System (Promega, USA). Each experiment was independently repeated three times.

### 2.13 Imiquimod-induced skin barrier damage in mice

All animal experiments were approved by the Animal Ethics Committee of Kunming Medical University and conducted in accordance with the Guide for the Care and Use of Laboratory Animals [(2020) Ethics Review L No. 13]. Sixteen male BALB/c mice (6–8 weeks old) were randomly divided into four groups: Normal (shaving + topical vaseline), Model (shaving + topical imiquimod), Antagomir-NC (shaving + injection of Antagomir-NC + topical imiquimod), and Antagomir (shaving + injection of Antagomir + topical imiquimod). Mice were first shaved. The model group received daily applications of imiquimod cream, while the normal group received vaseline as a control. For oligonucleotide injection in the experimental groups, Antagomir (5nmol/mouse) was injected into the dorsal skin for two consecutive days. From day 3 to day 13, mice received daily imiquimod cream applications, with additional Antagomir injections on days 6, 10, and 13. The total Antagomir dose was 25 nmol per mouse. The control group received Antagomir-NC injections (see Fig. 5a for detailed steps). On day 14, skin barrier function was assessed, and skin samples were collected for H&E staining, RT-qPCR, and immunofluorescence analysis.

### 2.14 Statistical analysis

Data analysis was performed using GraphPad Prism 7.0 software. Results are presented as means ± standard deviations. Inter-group comparisons were conducted using one-way analysis of variance (ANOVA), while multiple t-tests were employed for comparisons across multiple groups. Two-group comparisons were analyzed using t-tests. Statistical significance was defined as *∗P < 0.05*.

## 3 Results

### 3.1 Significant skin barrier damage in psoriasis patients

**Fig. 1:**
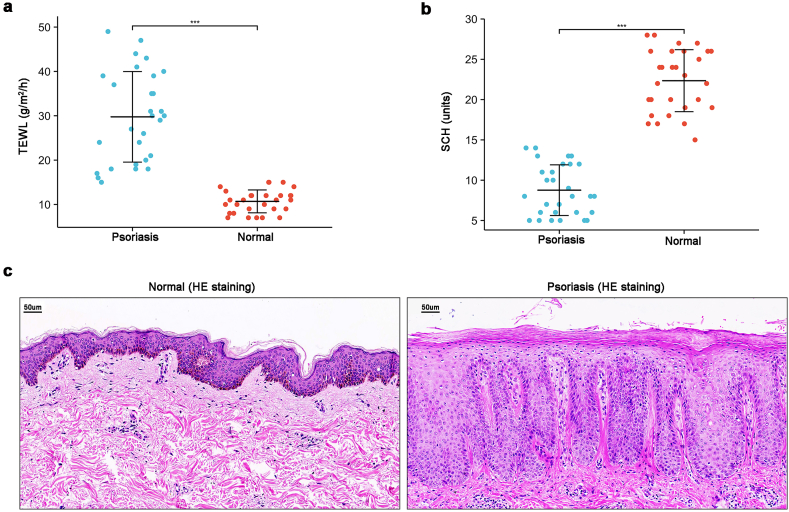
**Significant skin barrier damage in psoriasis patients** (a) Transepidermal water loss (TEWL) measurements (n = 29); (b) Stratum corneum hydration (SCH) measurements (n = 29); (c) H&E staining of normal and psoriatic tissues (5 normal vs 6 psoriasis). Data presented as mean ± SD. ∗∗∗*p<0.001*. Welch's *t*-test (a, b).

Psoriasis patients exhibited markedly increased Transepidermal Water Loss (TEWL) and decreased Stratum Corneum Hydration (SCH) (Fig. 1a and b). Histological examination using Hematoxylin-Eosin (HE) staining revealed significant epidermal thickening with pronounced hyperkeratosis and parakeratosis in psoriatic lesions compared to normal skin (Fig. 1c). Munro microabscesses were frequently observed in the stratum corneum, while the granular layer, the primary site of tight junctions, was diminished or absent. These findings strongly indicate compromised skin barrier function in psoriasis.

### 3.2 CLDN8 downregulation in psoriasis disrupts the keratinocyte barrier

**Fig. 2:**
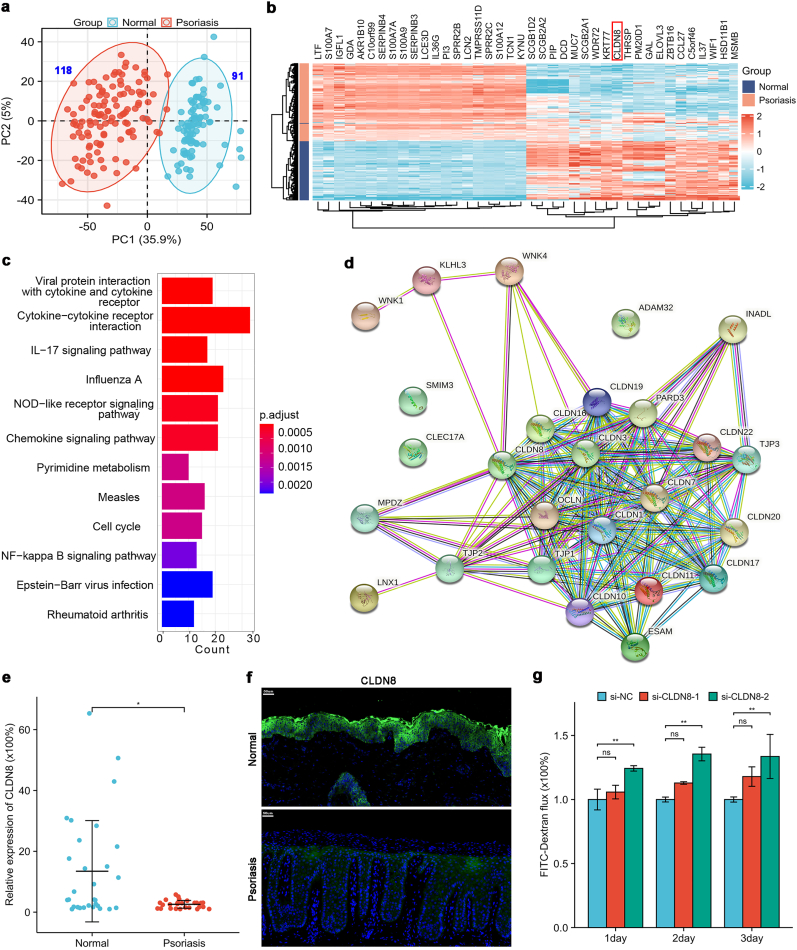
**CLDN8 reduction in psoriasis and its impact on keratinocyte barrier function** (a) PCA clustering analysis of psoriasis sequencing data (GSE78097, GSE14905, GSE13355; 91 normal vs 118 psoriasis); (b) Heat map of differentially expressed genes in psoriatic lesions (91 normal vs 118 psoriasis); (c) KEGG pathway enrichment of differentially expressed genes in psoriasis; (d) CLDN8 protein interaction network; (e) CLDN8 expression in tissues by RT-qPCR (31 normal vs 29 psoriasis); (f) CLDN8 expression in tissues by immunofluorescence (5 normal vs 6 psoriasis); (g) FITC-Dextran flux in keratinocytes after CLDN8 knockdown. Data presented as mean ± SD. ∗*p<0.05*, ∗∗*p<0.01*. ns: not significant. Wilcoxon rank-sum test (e); Kruskal-Wallis test (g).

Analysis of psoriasis sequencing datasets (GSE78097, GSE14905, GSE13355) from the NCBI database, comprising 91 normal tissues and 118 psoriatic skin lesions, revealed distinct clustering of normal and diseased samples through Principal Component Analysis (PCA) (Fig. 2a). Enrichment analysis demonstrated significantly reduced CLDN8 expression in psoriatic lesions, ranking 17th among differentially expressed genes with low expression (LogFC = −2.36, *P<0.05*) (Fig. 2b). KEGG pathway analysis highlighted the involvement of inflammatory signaling and NF-κB pathways (Fig. 2c), consistent with psoriatic phenotypes. CLDN8's protein interaction network, constructed using the STRING database, showed interactions with various tight junction proteins, including CLDN1 and TJP (Fig. 2d). RT-qPCR and immunofluorescence studies confirmed decreased CLDN8 expression in psoriatic skin (Fig. 2e and f). Furthermore, CLDN8 knockdown in keratinocytes resulted in significant barrier dysfunction, as evidenced by increased FITC-Dextran flux (Fig. 2g,supplemental figure c).

### 3.3 Hsa-miR-31-3p upregulation in psoriasis disrupts the keratinocyte barrier

**Fig. 3:**
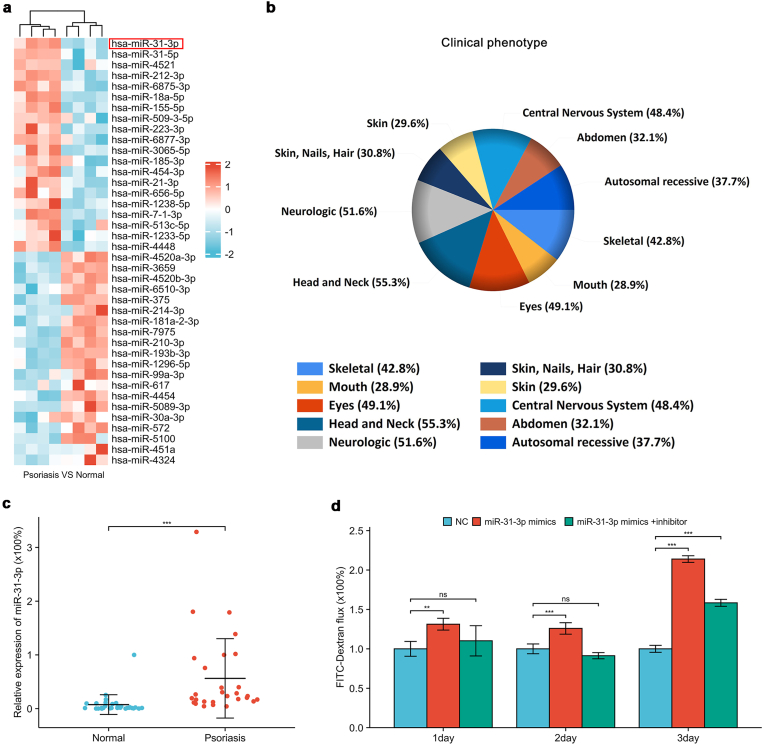
**Elevated hsa-miR-31-3p in psoriasis and its effect on keratinocyte barrier** (a) Heat map of differentially expressed miRNAs (GSE145305; 4 normal vs 4 psoriasis); (b) Differentially expressed miRNAs and associated clinical phenotypes; (c) Hsa-miR-31-3p expression in tissues by RT-qPCR (31 normal vs 29 psoriasis); (d) FITC-Dextran flux in keratinocytes after hsa-miR-31-3p overexpression. Data presented as mean ± SD. ∗∗*p<0.01*, ∗∗∗*p<0.001*. ns: not significant. Wilcoxon rank-sum test (c); One-way ANOVA (d).

Analysis of the GSE145305 dataset revealed significantly increased hsa-miR-31-3p expression in psoriatic lesions (Ranked first in high differential expression, LogFC = 5.78, *P<0.05*) (Fig. 3a). Differentially expressed miRNAs were found to account for 30.8 % of skin disease-related factors (Fig. 3b). RT-qPCR confirmed elevated hsa-miR-31-3p levels in psoriatic lesions (Fig. 3c). Overexpression of hsa-miR-31-3p in keratinocytes resulted in increased FITC-Dextran flux, indicating compromised barrier function (Fig. 3d).

### 3.4 Hsa-miR-31-3p disrupts skin barrier through targeting the 3′UTR of CLDN8

**Fig. 4:**
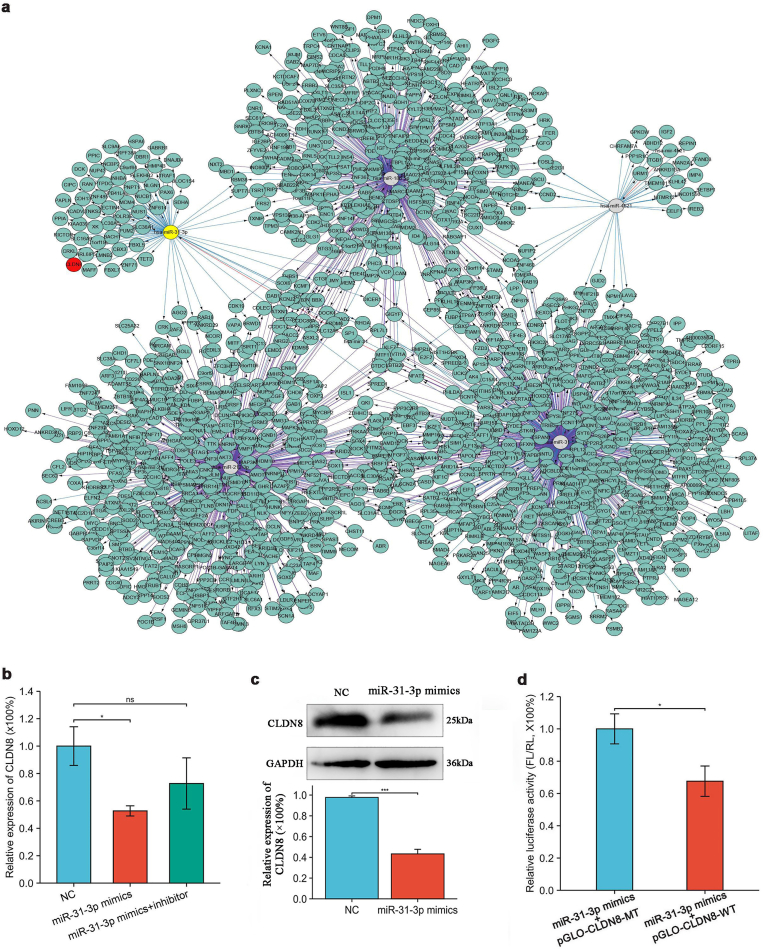
**Hsa-miR-31-3p targets CLDN8 3′UTR to disrupt skin barrier** (a) Interaction network between differentially expressed miRNAs and target genes; (b) CLDN8 mRNA expression by RT-qPCR after hsa-miR-31-3p overexpression; (c) CLDN8 protein expression by Western blot after hsa-miR-31-3p overexpression; (d) Dual-luciferase reporter gene assay. Data presented as mean ± SD. ∗*p<0.05*, ∗∗∗*p<0.001*. ns: not significant. One-way ANOVA (b, c); *t*-test (d).

Bioinformatics analysis predicted hsa-miR-31-3p binding to the CLDN8 3′UTR region, and a miRNA-target gene interaction network was constructed (Fig. 4a). Overexpression of hsa-miR-31-3p in keratinocytes significantly decreased CLDN8 expression, which was partially reversed by hsa-miR-31-3p inhibitor (Fig. 4b). Western blot analysis confirmed reduced CLDN8 protein levels following hsa-miR-31-3p overexpression (Fig. 4c). A dual-luciferase reporter assay validated the direct targeting of CLDN8 3′UTR by hsa-miR-31-3p (Fig. 4d, supplemental figure e). Additionally, hsa-miR-31-3p demonstrated an anti-apoptotic effect on keratinocytes, consistent with psoriatic hyperplastic lesions(See supplemental figure d).

### 3.5 Mmu-miR-31-3p antagomir ameliorates imiquimod-induced skin barrier damage in mice

**Fig. 5:**
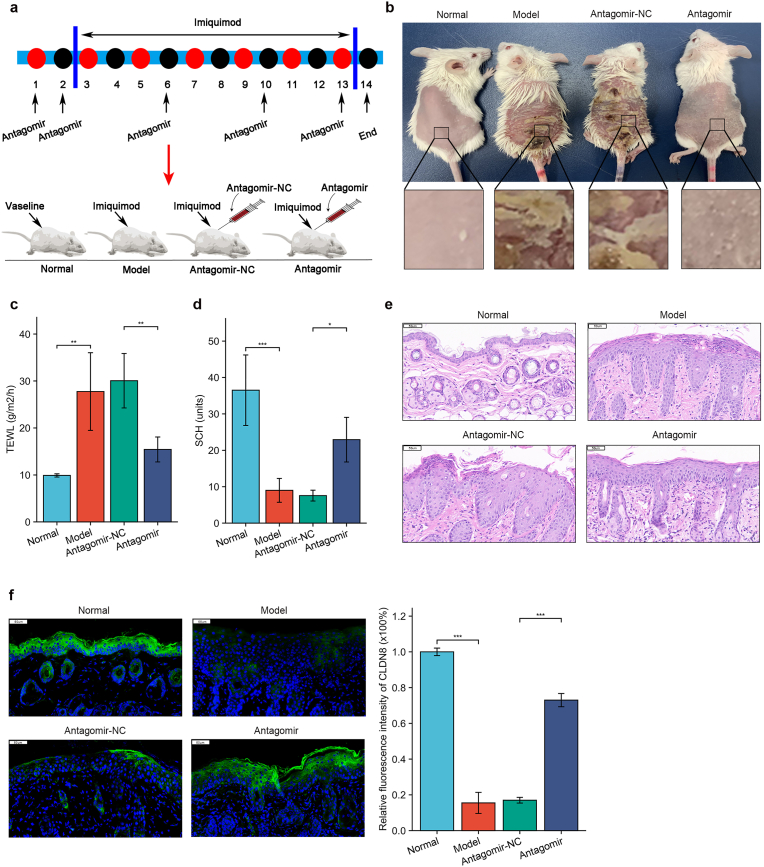
miR-31-3p antagomir repairs imiquimod-induced skin barrier damage in psoriatic mouse model (a) Schematic of animal experiments; (b) Mouse skin observations (n = 4); (c) TEWL measurements of mouse skin in each group (n = 4); (d) SCH measurements of mouse skin in each group (n = 4); (e) H&E staining of mouse skin in each group (n = 4); (f) Immunofluorescence detection of mouse skin in each group (n = 4). Data presented as mean ± SD. ∗*p<0.05*, ∗∗*p<0.01*, ∗∗∗*p<0.001*. ns: not significant. One-way ANOVA (c, d, f).

The therapeutic potential of mmu-miR-31-3p antagomir was evaluated in an imiquimod-induced psoriasis mouse model (Fig. 5a). Mice were divided into four groups: Normal, Model, Antagomir-NC and Antagomir. The Antagomir group showed reduced lesion area and improved skin appearance compared to the Model and Antagomir-NC groups (Fig. 5b). Non-invasive skin barrier assessments revealed significantly decreased TEWL and increased SCH in the Antagomir group (Fig. 5c and d). Histological examination demonstrated reduced epidermal thickening and improved skin morphology in the Antagomir group (Fig. 5e). Immunofluorescence analysis showed recovery of CLDN8 expression in the Antagomir group (Fig. 5f, supplemental figure f). These findings suggest that mmu-miR-31-3p antagomir can effectively repair imiquimod-induced skin barrier damage.

## 4 Discussion

This study provides novel insights into the mechanisms underlying skin barrier dysfunction in psoriasis. Our findings reveal a significant impairment of the skin barrier in psoriasis patients, characterized by increased transepidermal water loss (TEWL) and decreased stratum corneum hydration (SCH). These observations underscore the critical role of the skin barrier in psoriasis pathogenesis, a factor that has been historically underappreciated in clinical practice.

Central to our findings is the dysregulation of CLDN8, a key component of tight junctions, and hsa-miR-31-3p, a microRNA with emerging significance in skin disorders. Analysis of psoriasis transcriptome sequencing datasets revealed a marked decrease in CLDN8 expression coupled with a substantial increase in hsa-miR-31-3p levels within psoriatic lesions. This inverse relationship prompted further investigation into their potential interaction and functional consequences.

CLDN8, a 25 kDa epidermal secretory protein, plays a crucial role in maintaining the integrity of the skin barrier. It forms part of the physical barrier that regulates the paracellular passage of solutes and water between epithelial or endothelial cells [23,24]. Suárez-Fariñas et al. identified CLDN8 as a key barrier-related gene in atopic dermatis (AD) using RNA sequencing, highlighting its role in disease pathology and potential therapeutic implications [25]. Recent study showed that CLDN8 expression is significantly reduced in both lesional and nonlesional skin of pediatric AD patients, further emphasizing its importance in barrier integrity [11]. These studies collectively underscore the critical role of CLDN8 in skin diseases. Our findings align with these observations, protein interaction network analysis, using the String database, revealed that CLDN8 directly interacts with other tight junction proteins, including TJP1/ZO-1, TJP2/ZO-2, TJP3/ZO-3, CLDN4, and KLHL3. The significance of CLDN8 in barrier function was further confirmed through knockdown experiments, which demonstrated disruption of the keratinocyte permeability barrier.

The role of miRNAs in skin diseases has gained increasing attention due to their potential as diagnostic markers and therapeutic targets [13,14]. Our study focused on hsa-miR-31-3p, which we found to be significantly upregulated in psoriatic lesions (Ranked first in high differential expression). While previous research has primarily explored miR-31-5p, the role of hsa-miR-31-3p in psoriasis remained largely unexplored. Building on our previous work demonstrating hsa-miR-31-3p′s effect on the skin barrier in chronic actinic dermatitis through CLDN1 targeting [26]. we hypothesized a similar mechanism in psoriasis involving CLDN8 (Ranking 17th among differentially expressed genes with low expression).

Through a combination of bioinformatics analysis, RT-qPCR, Western blot, and dual-luciferase reporter gene assays, we established a direct targeting relationship between hsa-miR-31-3p and CLDN8. Functional studies demonstrated that both CLDN8 knockdown and hsa-miR-31-3p overexpression compromised the permeability barrier of keratinocytes, suggesting a mechanistic link between elevated hsa-miR-31-3p levels, reduced CLDN8 expression, and skin barrier dysfunction in psoriasis.

To validate our in vitro findings, we employed an imiquimod-induced psoriasis-like mouse model. Consistent with the clinical manifestation of psoriasis patient, these mice exhibited significant skin barrier impairment. Notably, injection of mmu-miR-31-3p antagomir into the back skin of these mice yielded promising results. We observed a significant reduction in inflammatory erythema, partial restoration of epidermal water loss and stratum corneum hydration, and recovery of CLDN8 expression as evidenced by immunofluorescence analysis.

In conclusion, These findings not only enhance our understanding of the molecular underpinnings of psoriasis but also present new avenues for therapeutic intervention. By targeting the hsa-miR-31-3p/CLDN8 pathway, it may be possible to restore skin barrier function in psoriasis patients, potentially improving disease outcomes and quality of life.

However, we have acknowledged the limitations of our study, including the sample size, which may affect the generalizability of our results. Future research should focus on translating these insights into clinical applications, exploring the potential of miRNA-based therapies in psoriasis management.

## CRediT authorship contribution statement

**Yunhua Tu:** Writing – review & editing, Writing – original draft, Project administration, Methodology, Investigation, Funding acquisition, Formal analysis, Data curation, Conceptualization. **Li Wang:** Writing – review & editing, Visualization, Validation, Investigation, Formal analysis, Data curation. **Lijun An:** Methodology, Investigation. **Li He:** Visualization, Validation, Supervision, Project administration.

## Data availability statement

The data supporting the findings of this study are available.

## Disclosure of interest

The authors declare that they have no known competing financial interests or personal relationships that could have appeared to influence the work reported in this paper.

## Declaration of competing interest

The authors declare that they have no known competing financial interests or personal relationships that could have appeared to influence the work reported in this paper.

## Figures and Tables

**Figure:**
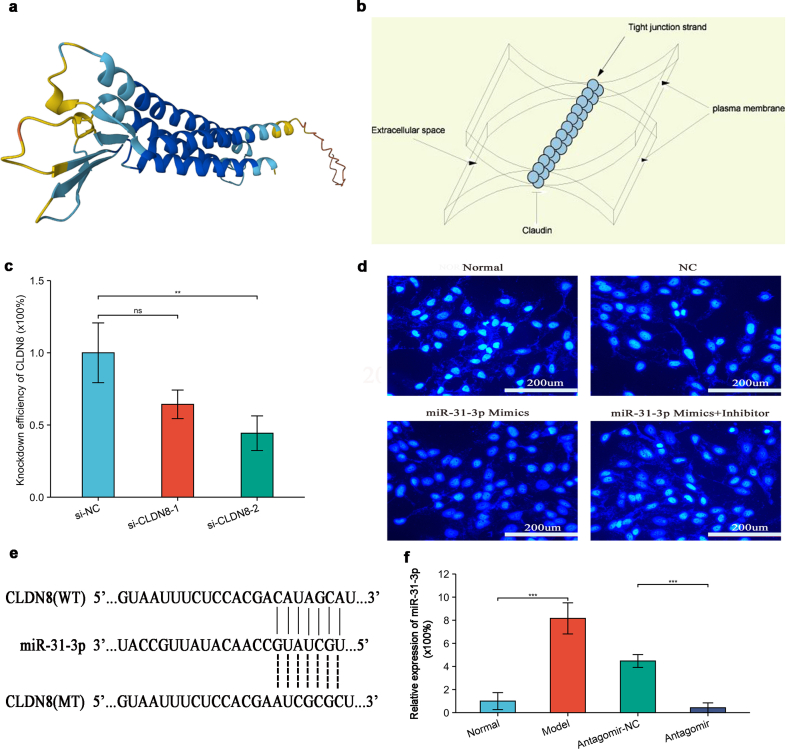
(a) CLDN8 protein structure; (b) Tight junction structure; (c) CLDN8 interference efficiency (n = 3); (d) Hoechst staining in each group; (e) Binding sites of hsa-miR-31-3p and CLDN8; (f) Relative expression of mmu-miR-31-3p in the mouse model (n = 3). Data presented as mean ± SD. ∗*p<0.05*, ∗∗*p<0.01*, ∗∗∗*p<0.001*. ns: not significant. One-way ANOVA (c); Paired sample *t*-test (f).

## Data Availability

Data will be made available on request.
