# Supplementary material for: Hsa-miR-31-3p targets CLDN8 to compromise skin barrier integrity in psoriasis
Source: Biochem Biophys Rep. 2025 Mar 13;42:101976. doi: 10.1016/j.bbrep.2025.101976 (PMC11954119; doi:10.1016/j.bbrep.2025.101976)
Supplement: Multimedia component 1 [file mmc1.docx]

**Supplementary Material**

| **RT-qPCR primers (5'-3')** | |
| --- | --- |
| H-CLDN8-forward | 5′- CTTCTGGGAAGGACTGTGGATG -3’ |
| H-CLDN8-reverse | 5′- GCCAAGAAGGACATCACGGAAG -3’ |
| M-CLDN8-forward | 5′- GTGGAGAGTGTCTGCCTTCATC -3’ |
| M-CLDN8-reverse | 5′- TAAGAGCCAGCAGGGAGTCGTA -3’ |
| Hsa-miR-31-3p | 5′- UGCUAUGCCAACAUAUUGCCAU-3’ |
| mmu-miR-31-3p | 5′- GCCtgctatgccaacatattgccatc -3’ |
| β-actin-forward | 5′-CACCATTGGCAATGAGCGGTTC-3’ |
| β-actin-reverse | 5′-AGGTCTTTGCGGATGTCCACGT-3’ |
| H-U6-forward | 5′-CTCGCTTCGGCAGCACA-3’ |
| Mmu-U6- forward | 5′- CTCGCTTCGGCAGCACA -3’ |
| GAPDH-forward | 5'- GCACCGTCAAGGCTGAGAAC-3' |
| GAPDH-reverse | 5'- TGGTGAAGACGCCAGTGGA-3' |

| **siRNA sequence** | |
| --- | --- |
| Si-CLDN8-NC | sense,5’- /rU//U//rC//rU//rC//rC//rG//rA//  A//rC//rG//rU//rG//rU//rC//rA//rC//rG//rU/TT-3’ ; antisense,5’-/rA//rC//rG//rU//rG//rA//rC//rA//r  C//rG//rU//rU//rC//rG//rG//rA//rG//rA//rA/TT-3’ |
| Si-CLDN8-1 | sense,5’-/rG//rG//rG//rA//rC//rA//rA//rU//r  G//rA//rG//rA//rA//rG//rG//rU//rG//rA//rA/TT-3’ ; antisense,5’-/rU//rU//rC//rA//rC//rC//rU//rU//rC//rU//rC//rA//rU//rU//rG//rU//rC//rC//rC/TT-3’ |
| SI-CLDN8-2 | sense,5’-/rG//rC//rU//rG//rA//rU//rU//rG//rU//rU//rG//rG//rA//rG//rG//rA//rG//rC//rU/TT-3’ ; antisense,5’-/rA//rG//rC//rU//rC//rC//rU//rC//rC//rA//rA//rC//rA//rA//rUl/rC//rA//rG//rC/TT-3’ |
| miR-31-3p mimics | sense,5’- UGCUAUGCCAACAUAUUGCCAU-3’ ; antisense,5’-GGCAAUAUGUUGGCAUAGCAUU-3’ |
| miR-31-3p inhibitor | 5′- AUGGCAAUAUGUUGGCAUAGCA -3’ |
| Mmu-miR-31-3p antagomir-NC | U*C*UACUCUUUCUAGGAGGUUGU*G*A-chol |
| Mmu-miR-31-3p antagomir | sense,5’- UGCUAUGCCAACAUAUUGCCAUC-3’ ; antisense,5’-G*A*UGGCAAUAUGUUGGCAUAG*C*A-chol-3’ |
| miR-31-3p mimics-NC | sense,5’- UUCUCCGAACGUGUCACGUTT -3’ ; antisense,5’- ACGUGACACGUUCGGAGAATT -3’ |
